# Enzyme and Biological Activities of the Water Extracts from the Plants *Aesculus hippocastanum*, *Olea europaea* and *Hypericum perforatum* That Are Used as Folk Remedies in Turkey

**DOI:** 10.3390/molecules25051202

**Published:** 2020-03-06

**Authors:** Cengiz Sarikurkcu, Marcello Locatelli, Angela Tartaglia, Vincenzo Ferrone, Aleksandra M. Juszczak, Mehmet Sabih Ozer, Bektas Tepe, Michał Tomczyk

**Affiliations:** 1 Department of Analytical Chemistry, Faculty of Pharmacy, Afyonkarahisar University of Health Sciences, Afyonkarahisar 03100, Turkey; sarikurkcu@gmail.com; 2 Department of Pharmacy, University of Chieti–Pescara “G. d’Annunzio”, 66100 Chieti, Italy; m.locatelli@unich.it (M.L.); angela.tartaglia@unich.it (A.T.); vincenzo.ferrone@unich.it (V.F.); 3 Department of Pharmacognosy, Faculty of Pharmacy with the Division of Laboratory Medicine, Medical University of Białystok, ul. Mickiewicza 2a, 15-230 Białystok, Poland; aleksandra.juszczak@umb.edu.pl; 4 Department of Chemistry, Faculty of Science and Literature, Manisa Celal Bayar University, Manisa 45140, Turkey; sabih.ozer@cbu.edu.tr; 5 Department of Molecular Biology and Genetics, Faculty of Science and Literature, Kilis 7 Aralik University, Kilis 79000, Turkey; bektastepe79@gmail.com

**Keywords:** phytochemical analysis, biological activities, *Aesculus hippocastanum*, *Olea europaea*, *Hypericum perforatum*, LC-ESI-MS/MS

## Abstract

Phenolic compounds are secondary metabolites that are found ubiquitously in plants, fruits, and vegetables. Many studies have shown that regular consumption of these compounds could have a positive effect on our health. The aim of this study was to compare the phytochemical contents of the water extracts from three different plants used as folk remedies in Turkey: *Aesculus hippocastanum*, *Olea europaea*, and *Hypericum perforatum*. A liquid chromatography-electrospray tandem mass spectrometry (LC-ESI-MS/MS) analysis was performed to explore the phenolic profiles. The biological activities of these extracts were also evaluated in terms of their antioxidant activities (2,2-diphenyl-1-picrylhydrazyl DPPH, 2,2′-azino-*bis*-(3-ethylbenzothiazoline-6-sulfonic acid ABTS, Ferric Reducing Antioxidant Power Assay FRAP, cupric ion reducing antioxidant capacity CUPRAC, β-carotene, phosphomolybdenum, and metal chelating) and enzyme inhibitory properties (against acetylcholinesterase, butyrylcholinesterase, and tyrosinase). The aqueous extract of *H. perforatum* showed the highest levels of total phenolic, flavonoid, and saponin contents. Protocatechuic acid, vanillic acid, verbascoside, hesperidin, hyperoside, apigenin 7-hexosides, and quercetin were the most common compounds found in this species. The results confirm that *A. hippocastanum*, *O. europaea*, and *H. perforatum* represent a potential source of natural-derived molecules with positive properties that could be used as valid starting point for new food supplements, and drugs in the pharmaceutical, cosmetic, and food industries.

## 1. Introduction

The use of natural extracts has gained a growing interest in recent years, and different foods, beverages, and cosmetics contain these extracts as functional ingredients [1]. Numerous studies have been conducted on plant components to identify the substances that are involved in the protection of human health, and phenolic compounds have been designated as the most important secondary metabolites. Polyphenols represent the largest group of organic compounds in plants, and more than 8000 different polyphenolic structures are known to date [2]. Among these polyphenol compounds, studies on flavonoids conducted both in vitro and in vivo have shown anti-inflammatory, anti-hypertensive, antioxidant, antithrombotic, and antiproliferative properties [3]. Although numerous synthetic drugs have been discovered, they are not without side effects on human health; for this reason, the use of natural compounds has been growing in recent years. Due to the popularity of olive oil (*Olea europaea* L.) in many different diets, particularly in Mediterranean cuisine, olive oil polyphenols have been extensively studied, and their beneficial effects on human health have now been consolidated [1]. In addition to oil, olive leaves, and their extracts are used in folk medicine to treat several diseases [4], and olive-leaf tea represents the most used among Mediterranean societies. The polyphenols present in olive leaves have been shown to possess important antioxidant, anti-inflammatory, antiatherogenic, and antimicrobial activities [5]. For this reason, interest in the potential health benefits of olive leaves has increased worldwide. *Aesculus hippocastanum* L. (Hippocastanaceae), commonly known as horse chestnut, represents another plant that is widely used in folk medicine to treat different diseases [6]. This plant is widely distributed in temperate climates, and its fruits or seeds have been traditionally used in the treatment of chronic venous insufficiency and inflammatory skin diseases [7]. More than 210 molecules were isolated from *A. hippocastanum* (e.g., triterpenoids, saponins, flavonoids, coumarins, carotenoids, and long fatty chain compounds) [8]. The genus *Hypericum* is the major between nine genera belonging to the family Clusiaceae, which has been used in traditional medicine thanks to their antidepressant, antimicrobial, and anti-inflammatory activities [9]. Due to the large use of *Hypericum* within traditional and modern medicine, many studies have been performed to better understand its composition. Even though the large amount of species in the genus *Hypericum*, the phytochemical and pharmacological activities of *H. perforatum*, commonly known as St. John’s Wort, have been the most studied. Currently, the efficacy of *H. perforatum* in the treatment of slight or modest depression has been validated, so that in Germany, *H. perforatum* is used as a substitute for conventional antidepressant drugs such as Prozac [10]. Even so, the biological properties of *H. perforatum* have been attributed to numerous bioactive compounds belonging to the classes of flavonoids and phenolic acids. These bioactive metabolites usually accumulate in leaves and flowers [11]. All of these species have received increasing interest in recent years due to the growing market demand for natural bioactive compounds that avoid the side effects that are typical of synthetic drugs. The objective of the current study was to determine the effects of the water extracts from the *A. hippocastanum* fruits, *O. europaea* leaves, and *H. perforatum* aerial parts by examining their enzyme inhibitory properties and antioxidant activities and correlating these results to their phytochemical profile based on chromatographic determination by liquid chromatography-electrospray tandem mass spectrometry (LC-ESI-MS/MS).

## 2. Results

### 2.1. Total Phenolic (TPC), Flavonoid (TFC), and Saponin (TSC) Contents

The selection of the extraction system covers a significant role in phytochemical analysis. Water is considered a better solvent than others when extracting phenolic compounds mostly because, in previous works, aqueous extracts showed higher efficiency and higher antioxidant activity than nonpolar extracts [12,13]. The results obtained for the water extracts of *O. europaea*, *H. perforatum*, and *A. hippocastanum* are shown in Table 1. Total phenolic (TPC), flavonoid (TFC), and saponin (TSC) are reported as gallic acid equivalents (GAEs), rutin equivalents (Res), and quillaja equivalents (QAEs), respectively. The aerial parts water extract of *H. perforatum* showed the highest values of TPC, TFC and TSC (181.02 ± 0.2 mg GAEs/g, 66.73 ± 0.22 mg REs/g, 336.31 ± 6.08 QAEs/g, respectively). The *O. europaea* leaf water extract presented a TPC of 92.15 ± 2.55 mg GAEs/g, TFC of 21.64 ± 1.76 mg REs/g, and TSC of 180.04 ± 5.80 QAEs/g. *A. hippocastanum* fruit water extract showed the lowest values of TPC (42.34 ± 0.38 mg GAEs/g) and TFC (11.69 ± 0.95 mg REs/g), but the TSC value was higher than that of the *O. europaea* extract (213.54 ± 11.81 QAEs/g).

### 2.2. Hyphenated Instrumental Analysis

The single molecules were identified by LC-MS/MS analysis [14,15]. *H. perforatum* contained the greatest number of phytochemical compounds (26 compounds), whereas the *O. europaea* and *A. hippocastanum* water extracts showed 24 phytochemical compounds each, as detected by this method. The protocatechuic acid results in all water extracts at high concentrations are as follows: 72.53 µg/mL, 761.67 µg/mL, and 176.08 µg/mL in *A. hippocastanum*, *H. perforatum*, and *O. europaea*, respectively. There were very high concentrations of hesperidin (3330.63 µg/mL), hyperoside (6164.64 µg/mL), and quercetin (3085.43 µg/mL) found in the *H. perforatum* extract. *O. europaea* showed high concentrations of verbascoside (4656.28 µg/mL), luteolin 7-hexosides (4185.59 µg/mL), and apigenin 7-hexosides (635.20 µg/mL). All of the results related to the quantification of the phytochemical compounds are shown in Table 2.

These natural compounds are generally broadly represented in the plant kingdom and show valuable activities related to their antioxidant actions, coupled to a possible enzyme inhibitory effect. As reported in the literature [16,17,18], these compounds are also well known to exert these functions not only through occasional uptake, but also specifically by means of a “chronic” and daily consumption in the diet.

### 2.3. Antioxidant Activities

The biological activities of natural compounds are intensely related to the antioxidant properties. Phenolic compounds play a vital role in neutralizing and inhibiting free radicals through scavenging or absorption. In this work, the biological activities of the *O. europaea*, *H. perforatum*, and *A. hippocastanum* extracts were evaluated with different assays likephosphomolybdenum, β-carotene bleaching, ferrous ion chelating, cupric ion reducing antioxidant capacity (CUPRAC), ferric reducing antioxidant power (FRAP), potassium ferricyanide), 2,2-azino-*bis*-3-ethylbenzothiazoline-6-sulfonic acid (ABTS·+) radical cation, 1,1-diphenyl-2-picrylhydrazyl (DPPH) radical, nitric oxide (·NO) radical, superoxide anion (O_2_^−^) radical, and hydroxyl (HO·) radical. Table 3 shows the results of the ferrous ion chelating and total antioxidant activity assays determined by the phosphomolybdenum and β-carotene bleaching methods. Although all extracts exhibited antioxidant activity, the assays showed that the *O. europaea* extract was the most efficient and presented the highest antioxidant capacity, except for in the ferrous ion chelating assay, which showed the highest value from the *H. perforatum* extract.

The radical scavenging activities of DPPH, ABTS, the superoxide anion, hydroxyl, and nitric oxide radicals were also evaluated. The extracts showed moderate activity toward the radicals, as reported in Table 4, except for the nitric oxide radical, where all the extracts showed moderate activity (6.53, 8.49, and 9.72 mmol butylated hydroxyanisole equivalents (BHAEs)/g extract for *O. europaea*, *H. perforatum*, and *A. hippocastanum*, respectively). Furthermore, the reducing power activities of the extracts were evaluated. The reducing power activities were studied by using potassium ferricyanide, CUPRAC, and FRAP tests (Table 5). The reducing power activity of the *H. perforatum* extract was found to be higher than those of the other extracts in all assays, with values of 137.03, 268.81, and 92.52 mg BHAEs/g extract for the potassium ferricyanide, CUPRAC, and FRAP tests, respectively.

### 2.4. Enzyme Inhibition Activities

Table 6 reports the inhibitory activities of the *O. europaea*, *H. perforatum*, and *A. hippocastanum* extracts against acetylcholinesterase (AChE), butyrylcholinesterase (BChE), and tyrosinase. According to the “cholinergic hypothesis” in patients of advanced age with Alzheimer’s disease (AD), dysfunction in neurons containing acetylcholine has been observed. For this reason, cholinesterase inhibitors represent a promising strategy for the treatment of AD, which represents to date the most common cause of cognitive impairment in the human population later in life [19]. The synthetic drugs currently used in AD have different limits and side effects, so the search for AChE inhibitors derived from plants has accelerated in recent years, and the benefits of these drugs are being studied not only for the treatment of AD, but also for other forms of dementia. The water extract from *O. europaea* showed the highest inhibition effect on BChE (107.21 mg galanthamine equivalents (GALAEs)/g extract), whereas the aqueous extracts of *H. perforatum* and *A. hippocastanum* showed low or no activity as cholinesterase inhibitors. Tyrosinase is a key enzyme in melanin biosynthesis. The increase in melanin levels may result in skin disorders such as melasma or skin cancer. In this regard, tyrosinase inhibitors play a key role in reducing excessive melanin synthesis [20]. All of the extracts showed inhibitory activity against tyrosinase, and the results were expressed as kojic acid equivalents (mg kojic acid equivalents (KAEs)/g extract). The *H. perforatum* extract was the most potent inhibitor with 28.89 mg KAEs/g extract compared with the *O. europaea* (24.28 mg KAEs/g extract) and *A. hippocastanum* extracts (19.59 mg KAEs/g extract). This high activity from the *H. perforatum* extract against tyrosinase may be due to its high amount of quercetin. Quercetin and other phenolic compounds have indeed shown inhibitory activity against this enzyme [21].

### 2.5. Correlation Coefficients between the Biological Activities

The correlation coefficients between the results of all of the experimental parameters are reported in Table 7, which shows the Pearson correlation coefficients. These coefficients are useful for comparing the statistical relationships between the parameters. There is a linear relationship between the total phenolic, flavonoid, and saponin content and many experimental assays. It is possible to observe an inverse relationship between the content of phytochemicals and the inhibitory activity of butyrylcholinesterase (BCIA), which is in agreement with data reported in Table 5 that showed the low activity of these extracts against this enzyme. Therefore, it could be hypothesized that the compounds responsible for cholinesterase inhibition have nonpolar characteristics. However, there are other interesting results that need to be highlighted: there is no positive correlation between the contents of verbascoside, luteolin 7-hexosides, apigenin 7-hexosides, and luteolin and the antioxidant activities. This suggests that the antioxidant activity cannot be directly linked to a single compound, and other compounds, most likely with a synergistic mechanism, could contribute to this activity, as suggested in another paper [22]. More studies on the phytochemical profile are required to recognize the natural bioactives in these species.

## 3. Discussion

### 3.1. Chemical Composition of the Extracts

To the best of our knowledge, in the literature, most of the studies on *O. europea* include data on the physical and chemical properties of the oils obtained from fruits and the chemical composition of these samples. The several works on the composition of extracts obtained from the leaves and aerial parts of this plant are limited. In one of these studies, the polyphenol content of three different varieties of *O. europea* was investigated and it was determined that the varieties contained 10.61%–24.06% of quercetin [23]. In another study, investigating the change in the physicochemical properties and chemical compositions of four different olive cultivars during fermentation, it was found that hydroxytyrosol, tyrosol, (+)−catechin, and quercetin were found to be the main compounds [24]. These data were found to be consistent with the phytochemical composition of *O. europea* presented in the current study.

Several works on the phytochemical composition of *H. perforatum* are reported in the literature. As can be seen from Table 2, as a result of the quantitative analysis, the extract was found to be rich in some of the phytochemicals given above. There are supporting studies in the literature that the *H. perforatum* contains hyperoside [25,26,27], hesperidin [28], quercetin [25,26,29,30], and chlorogenic acid [30,31,32] as the main components.

As can be seen from the section in which the phytochemical composition data of the present study is presented, the *A. hippocastanum* extract has been found to contain significant amounts of verbascoside, luteolin 7-hexosides, apigenin 7-hexosides, and protocatechuic acid. There are no reports in the literature that extracts from this plant contain verbascoside and protocatechuic acid. Therefore, the data presented regarding the phytochemicals in question is the first recorded for the literature. Although there is no literature data regarding the plant containing luteolin 7-hexosides and apigenin 7-hexosides, there is a study reporting that luteolin and apigenin are the main components [33].

### 3.2. Antioxidant Activity

Olive is a valuable product consumed by people for centuries. Since the oil of the fruits is very valuable, research has focused on oil samples. Therefore, there are few studies in the literature regarding the antioxidant activities of leaf samples. In one study, extracts from leaves were added to vacuum-packed salmon burgers and samples were kept at +4 °C for 16 days. It was found that lipid oxidation level decreased significantly in the samples during the cold storage period [34]. In another study investigating the antioxidant activity of natural and synthetic polyphenol derivatives in *O. europea*, it was determined that the antioxidant activity of lipophilic acetyl derivatives was higher [35]. In a study using a mixture of extracts obtained from *O. europea* leaves and *Hibiscus sabdariffa* flowers, the mixture was reported to show a high degree of cytoprotective and antioxidant activity on human umbilical vein endothelial (HUVECs) cells [36]. These data support the antioxidant activity findings obtained from the current study. Apart from these, some studies have been conducted on the antioxidant activities of olive oil samples [37,38,39]. However, since the examples used in the current study were not oils, these studies were left out of the discussion.

*H. perforatum* is one of the frequently researched species in terms of antioxidant activity potential. In a study investigating the change in the polyphenol content and antioxidant activity of tea prepared from *H. perforatum* depending on the infusion time, it was reported that the amount of dissolved hyperoside and chlorogenic increased as the infusion time increased and the antioxidant activity increased accordingly [40]. In a study investigating the effect of *H. perforatum* extract on brain oxidative stress markers, the extract was shown to exhibit remarkable antioxidant activity (decreased malondialdehyde (MDA) levels, increased catalase (CAT), and superoxide dismutase (SOD) activity) [41]. In a study examining the chemical composition, antioxidant and cytotoxic activity of eleven *Hypericum* species, *H. perforatum* was reported to exhibit excellent antioxidant/radical scavenging activity [42]. These data support the antioxidant activity findings obtained from the current study. Of course, it is possible to increase the number of samples related to the antioxidant activity of *H. perforatum*. However, since it is considered that it would be more reasonable to evaluate the current dated data, it is thought that there is no need to discuss other reports of a similar nature.

As can be seen from the tables in which antioxidant activity data are presented (Table 3, Table 4 and Table 5), the *A. hippocastanum* extract did not exhibit as high antioxidant activity as other extracts. There are also some studies in the literature regarding the antioxidant activity potential of this species. In a study investigating the antioxidant activity of *A. hippocastanum* mother tincture (TM), it was reported that the polyphenolic content of the sample was determined to be 506.8 mg GAEs/100 mL and exhibited significant antioxidant activity [43]. In another study evaluating the antioxidant activity of flower extracts obtained from *A. hippocastanum*, it was determined that the IC_50_ value measured as a result of the FRAP assay was 159.82 μg/mL and a dose-related decrease in reactive oxygen species production have been determined [6]. However, there are studies in the literature where the extract does not show high antioxidant activity. In a study investigating the antioxidant, anti-inflammatory, and venoconstrictor properties of *A. hippocastanum*, the highest activity was obtained against hydroxyl radicals (46.11%). The scavenging efficiency of the extract on superoxide radicals did not exceed 15% at pH 7.4 (at 100 μg/mL concentration) and the extract has been reported to be less effective on other radicals [44]. These data support the moderate antioxidant activity of *A. hippocastanum* presented in the current study.

### 3.3. Enzyme Inhibitory Activity

In the literature, there are some studies on the cholinesterase inhibitory activities of plant species evaluated in the herein reported work. The cholinesterase inhibitory activity of *O. europea* was studied only by one research group. In this study, where the phytochemical composition, antioxidant, and cholinesterase inhibitory activity of twenty-one different herbal samples were investigated *O. europea* did not show a significant inhibitory activity [45]. These data are consistent with the findings from the current study. In a study investigating the neuroprotective effect of ethyl acetate, methanol, and water extracts obtained from *H. perforatum*, it was found that the highest acetylcholinesterase inhibitory activity was exhibited by the methanol extract (49.54%). In addition, among the others, the ethyl acetate extract was found to show the best activity on butyrylcholinesterase (50.79%) [46]. Considering the data in Table 6, it can be seen that the water extract rich in polar compounds did not exhibit significant inhibitory activity on cholinesterases. According to the literature data, MeOH extract with medium polarity components and EtOAc extract with non-polar compounds showed higher cholinesterase inhibitory activity. This explains why the water extract showed low activity in the current study. As far as our literature survey could ascertain, no studies on the cholinesterase inhibitory activity of *A.* *hippocastanum* have been found in the literature. Therefore, data presented here regarding this plant could be assumed as the first record for the literature.

According to our literature survey, no report is available regarding the tyrosinase inhibitory activity of *O. europea* and *A. hippocastanum*. Therefore, it is considered that the data given here regarding the tyrosinase inhibitory activities of these plants may be the starting point for future studies. On the other hand, there are a few studies on the tyrosinase inhibitory activity of *H. perforatum*. In a study investigating the tyrosinase inhibitory activity of *H. perforatum*’s methanol extract, the extract was reported to exhibit 48.22% inhibitory activity. As a result of the phytochemical analysis, it was determined that the main components are chlorogenic acid, malic acid, quercitrin, and isoquercitrin, and tyrosinase inhibitory activity is closely related to the presence of these phenolics [31]. In another study investigating the tyrosinase inhibitory activities of ethyl acetate, methanol, and water extracts of *H. perforatum*, it was reported that only the methanol extract showed low activity (19.21%) [46]. Although it has been determined in the current study that *H. perforatum*’s tyrosinase inhibitor activity is higher than other extracts, it is useful to assume that the activity potential is actually at a moderate level. From this point of view, it can be seen that the findings obtained from the current study are compatible with the literature data.

## 4. Materials and Methods

### 4.1. Plant Materials

*Aesculus hippocastanum* L. fruits, *Olea europaea* L. leaves, and the aerial parts of *Hypericum perforatum* L. were collected from Manisa Province in the Aegean Region of Turkey in 2013, were deposited at the Department of Biology, Mugla Sitki Kocman University (Mugla, Turkey) under accession nos. OC.3425, OC.3480 and OC.3512, respectively, and authenticated by Dr. Olcay Ceylan. Legal permission has been obtained from the Ministry of Agriculture and Forestry (Turkey) to collect the plant samples from their natural habitat.

### 4.2. Preparation of the Extracts

The dried samples (10 g) were extracted by boiling in deionized water (200 mL) for 30 min. After filtration, the water extract was freeze-dried. All extracts were stored at +4 °C until being analyzed.

### 4.3. Antioxidant Activity

The antioxidant activities were carried out by using the experimental conditions reported by Zengin et al. [13] in order to better highlight the antioxidants, chelating, reducing power, and radical scavenging activities. For the phosphomolibdenum assay, a sample solution of 0.3 mL was added to 3 mL of 0.6 M sulfuric acid, 28 mM sodium phosphate, and 4 mM ammonium molybdate, and then the absorbance, at 695 nm, was read after a 90 min incubation at 95 °C.

For the β-carotene assay, 0.5 mg of β-carotene in 1 mL of chloroform was added to 25 µL of linoleic acid and 200 mg Tween 40. Then, after chloroform evaporation, 100 mL of oxygenated distilled water was added. This reaction solution was then added (1.5 mL) to test tubes and the sample solution (0.50 mL, 1 mg/mL). After an incubation of 2 h at 50 °C, the absorbances were read at 490 nm.

For the DPPH procedure, 1 mL of sample solution plus 4 mL of a 0.004% methanol solution of DPPH was incubated at room temperature for 30 min, and then the absorbances were read at 517 nm. A slightly similar procedure was applied for the ABTS assay. ABTS⋅+ solution, diluted with methanol to an absorbance of 0.700 ± 0.02 at 734 nm (2 mL) was added to 1 mL of the sample solution, and the absorbances were read at 734 nm after a 30 min incubation at room temperature.

For the ·NO assay, 0.5 mL of the sample solution mixed with 0.5 mL of sodium nitroprusside (5 mM) in phosphate buffer (0.2 M, pH 7.4) was incubated for 150 min at room temperature. To the incubated sample, Griess reagent was added, the solution was incubated for 30 min, and then the absorbances were read at 548 nm.

For O_2_·, a 0.25 mL of sample solution was added to a mixture of riboflavin (0.1 mL, 0.1 mg/mL), phosphate buffer (1 mL, 50 mM, pH 7.8), NBT (0.05 mL, 1 mg/mL), EDTA (ethylenediaminetetraacetic acid, 0.1 mL, 12 mM), and 1-butanol (0.5 mL). After an illumination time of 10 min at room temperature, the absorbances were read at 560 nm.

For HO·, 0.1 mL of ascorbic acid (1 mM), 0.28 mL of deoxyribose (10 mM), 0.41 mL of phosphate buffer (pH 7.4, 50 mM), 0.01 mL of ferric chloride (10 mM), 0.1 mL of hydrogen peroxide (10 mM), 0.1 mL of EDTA (0.1 mM), and finally 0.25 mL of sample solution were incubated for 12 h at 37 °C. The incubated sample, mixed with 0.75 mL trichloroacetic acid (2.8%, *w*/*v*) and 0.75 mL thiobarbituric acid reagent (1% *w*/*v*, in 50 mM NaOH), and heated at 100 °C for 1 h bring to the absorbance signals at 532 nm.

For the potassium ferricyanide assay, 0.5 mL of sample solution, 0.5 mL of phosphate buffer (0.2 M, pH 6.6), and 0.5 mL of potassium ferricyanide (1%), was incubated at 50 °C for 20 min. Then, after the addition of 0.5 mL of trichloroacetic acid (10%), 2.5 mL of deionized water, and 0.5 mL of ferric chloride (0.1%), the absorbances were read at 700 nm.

For the CUPRAC assay, 0.5 mL of sample solution, 1 mL of CuCl_2_ (10 mM), 1 mL of neocuproine (7.5 mM), and 1 mL of NH_4_Ac buffer (1 M, pH 7.0) was incubated for 30 min at room temperature and then the absorbances were read at 450 nm.

For the FRAP assay, 0.1 mL of sample solution, 2 mL of a reagent mixture (acetate buffer (0.3 M, pH 3.6), 2,4,6-*tris*(2-pyridyl)-*S*-triazine (TPTZ) (10 mM) in 40 mM HCl and ferric chloride (20 mM) in a ratio of 10:1:1 (*v*/*v*/*v*) was incubated at room temperature for 30 min and then the absorbances were read at 593 nm.

For ferrous ion chelating, 2 mL of sample solution, 0.05 mL of FeCl_2_ solution (2 mM), 0.2 mL of ferrozine (5 mM) was incubated at room temperature for 10 min and the absorbances were read at 562 nm.

### 4.4. Enzyme Inhibitory Activity

Enzymatic inhibitory activities were analyzed on acetylcholinesterase AChE, BChE, and tyrosinase by using the methods reported elsewhere [12].

For the AChE and BChE assay, 50 μL of sample solution, 125 μL of DTNB, and 25 μL of AChE (or BChE) in Tris–HCl buffer (pH 8.0) was placed in a 96-well microplate and incubated for 15 min at 25 °C. Through the addition of 25 μL of acetylthiocholine iodide (ATCI) or butyrylthiocholine chloride (BTCl), the reaction was initiated. The absorbance, after a 10 min incubation at 25 °C, was read at 405 nm.

For the tyrosinase assay, 25 μL of sample solution, 40 μL of tyrosinase solution, and 100 μL of phosphate buffer (pH 6.8) in a 96-well microplate was incubated for 15 min at 25 °C. By the addition of 40 μL of L-DOPA the reaction was initiated. The absorbance, after a 10 min incubation at 25 °C, was read at 492 nm.

### 4.5. Total Bioactive Components

Total phenolic, flavonoid, and saponin contents were determined by employing methods given in the literature [47].

### 4.6. Liquid Chromatography-Electrospray Tandem Mass Spectrometry (LC-ESI-MS/MS) Analysis

An Agilent Technologies 1260 Infinity liquid chromatography system coupled to a 6420 Triple Quadrupole mass spectrometer was used for the qualitative and quantitative analyses. The separation was achieved by means of a Poroshell 120 EC-C18 (100 mm × 4.6 mm I.D., 2.7 μm) column, and using 0.1% formic acid (A)/methanol (B) as mobile (selected based on the sufficient chromatographic resolution of isomeric compounds). This configuration also allows for improvements in the method sensitivity. The gradient elution profile herein adopted was set as follows: 0.00 min 2% B eluent, 3.00 min 2% B eluent, 6.00 min 25% B eluent, 10.00 min 50% B eluent, 14.00 min 95% B eluent, 17.00 min 95% B, and 17.50 min 2% B eluent. The column temperature was maintained at 25 °C. The flow rate was 0.4 mL/min, and the injection volume was 2.0 μL. The MS instrumentation was directly coupled with the LC system via an ESI source, operating both in negative and positive multiple reaction monitoring (MRM) mode. The ESI parameters were: capillary voltage −3.5 kV, gas temperature 300 °C, gas flow 11 mL/min, nebulizer pressure 40 psi.

The different analytes were identified by means of their retention times, mass spectra, and tandem mass spectra. Specifically, quantitative analyses were performed using a specific MRM transition for each analyte.

### 4.7. Statistical Analysis

All of the analyses were carried out in triplicate, and the results were reported as mean and standard deviation (mean ± SD). Additionally, the ANOVA and Tukey’s honestly significant difference post hoc test (α = 0.05) were performed using SPSS v. 22.0 software to determine the differences between the assays. A further check to evaluate the association between the results was determined by means of Pearson’s linear correlation.

## 5. Conclusions

In the present work, the phenolic, flavonoid, and saponin contents of *A. hippocastanum*, *O. europaea,* and *H. perforatum* have been reported. Furthermore, the antioxidant activity and the enzymatic inhibitory activity against AChE, BChE, and tyrosinase were reported. All extracts showed high contents of phytochemical compounds and important antioxidant and enzymatic inhibitory activities. The results of the reported work support the beneficial utilization of these plants as powerful natural antioxidants that prevent the excessive production of free radicals and possess enzyme inhibitory activities against tyrosinase. These results are added to the numerous preliminary studies that report the beneficial effects of these natural compounds for the prevention and treatment of many diseases. However, more in vivo studies and clinical trials are still needed.

## Figures and Tables

**Table 1:** Total phenolic, flavonoid, and saponin contents of the water extracts from plants.

Samples	TPC (mg GAEs/g) ^y^	TFC (mg REs/g) ^z^	TSC (QAEs/g) ^w^
*O. europaea*	92.15 ± 2.55	21.64 ± 1.76	180.04 ± 5.80
*H. perforatum*	181.02 ± 1.47	66.73 ± 0.22	336.31 ± 6.08
*A. hippocastanum*	42.34 ± 0.38	11.69 ± 0.95	213.54 ± 11.81

^y^ GAEs, gallic acid equivalents; ^z^ REs, rutin equivalents; ^w^ QAEs, quillaja equivalents.

**Table 2:** Concentration of the identified compounds in the water extracts ^x^.

Compounds	Class	Sub-Class	Concentration (µg/g Extract)		
			*A. hippocastanum*	*H. perforatum*	*O. europaea*
Gallic acid	Phenolic acids	Hydroxybenzoic acids	8.92 ± 0.18 ^b^	79.64 ± 1.14 ^a^	5.80 ± 0.01 ^c^
Protocatechuic acid	Phenolic acids	Hydroxybenzoic acids	72.53 ± 0.38 ^c^	761.67 ± 8.00 ^a^	176.08 ± 3.06 ^b^
3,4-Dihydroxyphenylacetic acid	Phenolic acids	Hydroxyphenylacetic acids	Nd ^y^	4.44 ± 0.17 ^b^	6.17 ± 0.36 ^a^
(+)-Catechin	Flavonoids	Flavanols	3.24 ± 0.10 ^b^	62.69 ± 2.25 ^a^	nd
Pyrocatechol	Other polyphenols	Other polyphenol	nd	7.70 ± 0.72 ^b^	10.26 ± 0.01 ^a^
Chlorogenic acid	Phenolic acids	Hydroxycinnamic acids	8.00 ± 0.14 ^c^	2131.99 ± 32.75 ^a^	11.02 ± 0.16 ^b^
2,5-Dihydroxybenzoic acid	Phenolic acids	Hydroxybenzoic acids	nd	33.26 ± 0.06 ^a^	11.75 ± 0.41 ^b^
4-Hydroxybenzoic acid	Phenolic acids	Hydroxybenzoic acids	13.84 ± 0.03 ^c^	102.16 ± 0.93 ^a^	36.28 ± 0.33 ^b^
(-)-Epicatechin	Flavonoids	Flavanols	nd	118.83 ± 0.92	nd
Vanillic acid	Phenolic acids	Hydroxybenzoic acids	9.04 ± 0.77 ^c^	327.80 ± 7.33 ^a^	41.52 ± 1.16 ^b^
Caffeic acid	Phenolic acids	Hydroxycinnamic acids	2.75 ± 0.03 ^c^	14.10 ± 0.07 ^a^	6.90 ± 0.10 ^b^
Syringic acid	Phenolic acids	Hydroxybenzoic acids	24.63 ± 1.48 ^a^	23.27 ± 1.86 ^a^	nd
3-Hydroxybenzoic acid	Phenolic acids	Hydroxybenzoic acids	nd	nd	nd
Vanillin	Other polyphenols	Hydroxybenzaldehydes	nd	nd	nd
Verbascoside	Phenolic acids	Hydroxycinnamic acids	15.36 ± 0.06 ^b^	10.66 ± 0.19 ^c^	4656.28 ± 114.23 ^a^
Taxifolin	Flavonoids	Dihydroflavonols	1.31 ± 0.01 ^c^	42.63 ± 0.17 ^a^	3.22 ± 0.29 ^b^
Sinapic acid	Phenolic acids	Hydroxycinnamic acids	7.52 ± 0.15	nd	nd
p-Coumaric acid	Phenolic acids	Hydroxycinnamic acids	10.62 ± 0.07 ^b^	45.06 ± 0.41 ^a^	44.12 ± 0.71 ^a^
Ferulic acid	Phenolic acids	Hydroxycinnamic acids	2.94 ± 0.15 ^c^	27.08 ± 0.19 ^a^	9.56 ± 0.25 ^b^
Luteolin 7-hexosides	Flavonoids	Flavones	3.82 ± 0.05 ^c^	9.33 ± 0.20 ^b^	4185.59 ± 85.81 ^a^
Hesperidin	Flavonoids	Flavanones	40.25 ± 2.19 ^c^	3330.63 ± 19.56 ^a^	57.16 ± 2.17 ^b^
Hyperoside	Flavonoids	Flavonols	203.66 ± 2.50 ^b^	6164.64 ± 0.73 ^a^	10.78 ± 0.67 ^c^
Rosmarinic acid	Phenolic acids	Hydroxycinnamic acids	16.90 ± 0.70 ^b^	64.82 ± 0.02 ^a^	5.91 ± 0.45 ^c^
Apigenin 7-hexosides	Flavonoids	Flavones	4.65 ± 0.07 ^b^	2.39 ± 0.37 ^c^	635.20 ± 14.68 ^a^
2-Hydroxycinnamic acid	Phenolic acids	Hydroxycinnamic acids	nd	nd	nd
Pinoresinol	Lignans	Lignans	14.20 ± 1.10 ^a^	6.64 ± 1.52 ^b^	15.57 ± 0.13 ^a^
Eriodictyol	Flavonoids	Flavanones	1.63 ± 0.04 ^c^	5.48 ± 0.17 ^a^	2.70 ± 0.01 ^b^
Quercetin	Flavonoids	Flavonols	51.15 ± 0.50 ^b^	3085.43 ± 124.60 ^a^	14.011 ± 3.23 ^c^
Luteolin	Flavonoids	Flavones	4.85 ± 0.30 ^b^	5.57 ± 0.14 ^b^	97.29 ± 0.18 ^a^
Kaempferol	Flavonoids	Flavonols	7.06 ± 0.74 ^b^	27.91 ± 4.65 ^a^	2.58 ± 0.01 ^c^
Apigenin	Flavonoids	Flavones	4.69 ± 0.14 ^b^	nd	14.78 ± 0.11 ^a^

^x^ Different subscripts (a, b, c) in the same row indicate significant difference (*p* < 0.05); ^y^ nd, not detected.

**Table 3:** Ferrous ion chelating, phosphomolybdenum, and β-carotene bleaching activities of the water extracts from plants ^x^.

Samples	β-Carotene Bleaching (%) ^y^	Phosphomolybdenum (mg AAEs/g Extracts) ^z^	Ferrous Ion Chelating (mg EDTAEs/g Extracts) ^z^
*O. europaea*	78.86 ± 3.03 ^b^	161.20 ± 8.23 ^a^	33.26 ± 0.48 ^c^
*H. perforatum*	77.00 ± 0.32 ^b^	145.13 ± 4.69 ^b^	75.35 ± 0.48 ^a^
*A. hippocastanum*	32.08 ± 4.66 ^c^	74.47 ± 3.93 ^c^	49.72 ± 4.29 ^b^
BHA	95.78 ± 1.13 ^a^	-	-
BHT	95.31 ± 0.56 ^a^	-	-

^x^ Different subscripts in the same column indicate significant difference (*p* < 0.05, abc); ^y^ Given as percentage of % inhibition of the linoleic acid at 2 mg/mL concentration; ^z^ AAEs, ascorbic acid equivalents; ^z^ EDTAEs, ethylenediaminetetraacetic acid (disodium salt) equivalents.

**Table 4:** Radical scavenging action of the water extracts from plants ^x^.

Assays	Radical Scavenging Action (mmol BHAEs/g Extract) ^y^		
	*O. europaea*	*H. perforatum*	*A. hippocastanum*
DPPH radical	0.41 ± 0.01 ^b^	0.90 ± 0.01 ^a^	0.11 ± 0.01 ^c^
ABTS radical cation	0.72 ± 0.05 ^b^	1.40 ± 0.08 ^a^	0.38 ± 0.02 ^c^
Superoxide anion	1.11 ± 0.01 ^a^	1.05 ± 0.01 ^b^	0.76 ± 0.02 ^c^
Hydroxyl radical	1.17 ± 0.03 ^a^	0.90 ± 0.07 ^b^	0.66 ± 0.02 ^c^
Nitric oxide radical	6.53 ± 0.71 ^a^	8.49 ± 1.28 ^a^	9.72 ± 0.18 ^a^

^x^ Different subscripts in the same row indicate significant difference (*p* < 0.05, abc); ^y^ BHAEs, butylated hydroxyanisole equivalents.

**Table 5:** Reducing power action of the water extracts from plants ^x^.

Samples	Reducing Power (mg BHAEs/g Extract) ^y^		
	Potassium Ferricyanide	CUPRAC	FRAP
*O. europaea*	89.60 ± 0.28 ^b^	182.01 ± 3.30 ^b^	85.49 ± 0.02 ^b^
*H. perforatum*	137.03 ± 0.09 ^a^	268.81 ± 5.32 ^a^	92.52 ± 0.11 ^a^
*A. hippocastanum*	20.12 ± 2.25 ^c^	59.01 ± 0.37 ^c^	29.53 ± 1.14 ^c^

^x^ Different subscripts in the same column indicate significant difference (*p* < 0.05, abc); ^y^ BHAEs, butylated hydroxyanisole equivalents.

**Table 6:** Enzyme inhibitory activities of the water extracts from plants ^x^.

Assays	*O. europaea*	*H. perforatum*	*A. hippocastanum*
AChE (mg GALAEs/g extract) ^y^	na ^w^	na	na
BChE (mg GALAEs/g extract) ^y^	107.21 ± 7.78 ^a^	6.39 ± 0.58 ^b^	na
Tyrosinase (mg KAEs/g extract) ^z^	24.28 ± 0.94 ^b^	28.89 ± 1.63 ^a^	19.59 ± 1.62 ^c^

^x^ Different subscripts in the same row indicate significant difference (*p* < 0.05, abc); ^y^ GALAEs, galanthamine equivalents; ^z^ KAEs, kojic acid equivalents; ^w^ na, not active.

**Table 7:** Correlation coefficients between the assays ^x^.

	TACB	TAP	MCA	DPPH	ABTS	SAR	HR	NOR	PFRP	CUPRAC	FRAP	BCIA	TIA
Total phenolic	0.752	0.653	0.724	0.999^y^	0.999^y^	0.663	0.323	−0.229	0.964	0.966	0.835	−0.108	0.986
Total flavonoid	0.612	0.496	0.842	0.977	0.987	0.508	0.136	−0.039	0.895	0.899	0.715	−0.296	0.937
Total saponin	0.280	0.143	0.981	0.831	0.858	0.157	−0.237	0.330	0.670	0.676	0.408	−0.625	0.743
Gallic acid	0.502	0.376	0.907	0.940	0.956	0.389	0.003	0.094	0.828	0.833	0.615	−0.420	0.882
Protocatechuic acid	0.342	0.207	0.967	0.865	0.890	0.221	−0.173	0.268	0.717	0.723	0.467	−0.573	0.785
Chlorogenic acid	0.468	0.340	0.922	0.926	0.945	0.354	−0.035	0.132	0.806	0.811	0.585	−0.454	0.863
4-Hydroxybenzoic acid	0.239	0.101	0.989	0.806	0.836	0.115	−0.277	0.369	0.638	0.645	0.370	−0.657	0.714
Vanillic acid	0.385	0.253	0.954	0.888	0.911	0.267	−0.126	0.222	0.749	0.755	0.508	−0.534	0.813
Verbascoside	−0.999 ^y^	−0.985	−0.126	−0.790	−0.757	−0.987	−0.848	0.793	−0.915	−0.912	−0.995	−0.545	−0.869
Luteolin 7-hexosides	−0.999 ^y^	−0.985	−0.124	−0.788	−0.755	−0.987	−0.849	0.794	−0.915	−0.911	−0.995	−0.546	−0.868
Hesperidin	0.465	0.337	0.923	0.925	0.943	0.351	−0.038	0.135	0.804	0.810	0.582	−0.457	0.861
Hyperoside	0.493	0.367	0.911	0.937	0.954	0.380	−0.006	0.104	0.823	0.828	0.608	−0.429	0.877
Rosmarinic acid	0.617	0.501	0.839	0.978	0.988	0.513	0.142	−0.045	0.898	0.902	0.719	−0.290	0.939
Apigenin 7-hexosides	−0.999 ^y^	−0.984	−0.128	−0.791	−0.758	−0.987	−0.847	0.791	−0.916	−0.913	−0.995	−0.543	−0.870
Quercetin	0.479	0.351	0.918	0.930	0.948	0.365	−0.023	0.121	0.813	0.818	0.594	−0.444	0.869
Luteolin	−0.999 ^y^	−0.986	−0.118	−0.785	−0.751	−0.988	−0.852	0.797	−0.912	−0.909	−0.994	−0.551	−0.865
Kaempferol	0.609	0.492	0.845	0.976	0.986	0.505	0.132	−0.035	0.893	0.898	0.712	−0.300	0.935

^x^ Data represents the Pearson correlation coefficient R. (TACB: total antioxidant activity by β-carotene bleaching method; TAP: total antioxidant activity by phosphomolybdenum method; MCA: metal chelating activity; DPPH: DPPH radical scavenging activity; ABTS: ABTS radical scavenging activity; SAR: superoxide anion radical scavenging activity; HR: hydroxyl radical scavenging activity; NOR: nitric oxide radical scavenging activity; CUPRAC: CUPRAC reducing power potential; FRAP: FRAP reducing power potential; PFRP: potassium ferricyanide reducing power potential; BCIA: butyrylcholinesterase inhibitory activity; TIA: Tyrosinase inhibitory activity; TSC: total saponin content; TPC: total phenolic content; TFC: total flavonoid content); ^y^ indicates *p* < 0.05.

## References

[1] Komes D., Belscak-Cvitanovic A., Horzic D., Rusak G., Likic S., Berendika M. (2011). Phenolic composition and antioxidant properties of some traditionally used medicinal plants affected by the extraction time and hydrolysis. Phytochem. Anal..

[2] Gorzynik-Debicka M., Przychodzen P., Cappello F., Kuban-Jankowska A., Marino Gammazza A., Knap N., Wozniak M., Gorska-Ponikowska M. (2018). Potential health benefits of olive oil and plant polyphenols. Int. J. Mol. Sci..

[3] Benayad Z., Martinez-Villaluenga C., Frias J., Gomez-Cordoves C., Es-Safi N.E. (2014). Phenolic composition, antioxidant and anti-inflammatory activities of extracts from Moroccan *Opuntia ficus-indica* flowers obtained by different extraction methods. Ind. Crops Prod..

[4] Difonzo G., Russo A., Trani A., Paradiso M.V., Ranieri M., Pasqualone A., Summo C., Tamma G., Silletti R., Caponio F. (2017). Green extracts from Coratina olive cultivar leaves: Antioxidant characterization and biological activity. J. Funct. Foods.

[5] Herrero M., Temirzoda T.N., Segura-Carretero A., Quirantes R., Plaza M., Ibanez E. (2011). New possibilities for the valorization of olive oil by-products. J. Chromatogr. A.

[6] Dudek-Makuch M., Studzińska-Sroka E., Korybalska K., Czepulis N., Łuczak J., Rutkowski R., Marczak Ł., Długaszewska J., Grabowska K., Stobiecki M. (2019). Biological activity of *Aesculus hippocastanum* flower extracts on vascular endothelial cells cultured in vitro. Phytochem. Lett..

[7] Kędzierski B., Kukula-Koch W., Widelski J., Głowniak K. (2016). Impact of harvest time of *Aesculus hippocastanum* seeds on the composition, antioxidant capacity and total phenolic content. Ind. Crops Prod..

[8] Zhang Z., Li S., Lian X., Temple A., State S.F.A. (2010). An overview of genus *Aesculus* L.: Ethnobotany phytochemistry, and pharmacological activities. Pharm. Crop..

[9] Dresler S., Kovacik J., Strzemski M., Sowa I., Wojciak-Kosior M. (2018). Methodological aspects of biologically active compounds quantification in the genus *Hypericum*. J. Pharm. Biomed. Anal..

[10] Wise K., Selby-Pham S., Bennett L., Selby-Pham J. (2019). Pharmacokinetic properties of phytochemicals in *Hypericum perforatum* influence efficacy of regulating oxidative stress. Phytomedicine.

[11] Tusevski O., Gadzovska Simic S., Krstikj M., Petreska Stanoeva J., Stefova M. (2018). Phenolic profile and biological activity of *Hypericum perforatum* L.: Can roots be considered as a new source of natural compounds?. S. Afr. J. Bot..

[12] Sarikurkcu C., Zengin G., Oskay M., Uysal S., Ceylan R., Aktumsek A. (2015). Composition, antioxidant, antimicrobial and enzyme inhibition activities of two *Origanum vulgare* subspecies (subsp. *vulgare* and subsp. *hirtum*) essential oils. Ind. Crops Prod..

[13] Zengin G., Sarikurkcu C., Uyar P., Aktumsek A., Uysal S., Kocak M.S., Ceylan R. (2015). *Crepis foetida* L. subsp. *rhoeadifolia* (Bieb.) Celak. as a source of multifunctional agents: Cytotoxic and phytochemical evaluation. J. Funct. Foods.

[14] Cittan M., Celik A. (2018). Development and validation of an analytical methodology based on liquid chromatography-electrospray tandem mass spectrometry for the simultaneous determination of phenolic compounds in olive leaf extract. J. Chromatogr. Sci..

[15] Tlili N., Kirkan B., Sarikurkcu C. (2019). LC-ESI-MS/MS characterization, antioxidant power and inhibitory effects on α-amylase and tyrosinase of bioactive compounds from hulls of *Amygdalus communis*: The influence of the extracting solvents. Ind. Crops Prod..

[16] Fardet A., Chardigny J.M. (2013). Plant-Based foods as a source of lipotropes for human nutrition: A survey of in vivo studies. Crit. Rev. Food Sci. Nutr..

[17] Guarrera P.M., Savo V. (2016). Wild food plants used in traditional vegetable mixtures in Italy. J. Ethnopharmacol..

[18] Venkidasamy B., Selvaraj D., Nile A.S., Ramalingam S., Kai G., Nile S.H. (2019). Indian pulses: A review on nutritional, functional and biochemical properties with future perspectives. Trends Food Sci. Technol..

[19] Mukherjee P.K., Kumar V., Mal M., Houghton P.J. (2007). Acetylcholinesterase inhibitors from plants. Phytomedicine.

[20] Mocan A., Diuzheva A., Badarau S., Moldovan C., Andruch V., Carradori S., Campestre C., Tartaglia A., De Simone M., Vodnar D. (2019). Liquid phase and microwave-assisted extractions for multicomponent phenolic pattern determination of five Romanian *Galium* species coupled with bioassays. Molecules.

[21] Kim Y.-J., Uyama H. (2005). Tyrosinase inhibitors from natural and synthetic sources: Structure, inhibition mechanism and perspective for the future. Cell. Mol. life Sci..

[22] Gonçalves S., Grevenstuk T., Martins N., Romano A. (2015). Antioxidant activity and verbascoside content in extracts from two uninvestigated endemic *Plantago* spp.. Ind. Crops Prod..

[23] Nazzaro F., Fratianni F., Cozzolino R., Martignetti A., Malorni L., De Feo V., Cruz A.G., D’acierno A. (2019). Antibacterial activity of three extra virgin olive oils of the campania region, southern italy, related to their polyphenol content and composition. Microorganisms.

[24] Kiai H., Hafidi A. (2014). Chemical composition changes in four green olive cultivars during spontaneous fermentation. LWT Food Sci. Technol..

[25] Chen H., Muhammad I., Zhang Y., Ren Y., Zhang R., Huang X., Diao L., Liu H., Li X., Sun X. (2019). Antiviral activity against infectious bronchitis virus and bioactive components of *Hypericum perforatum* L.. Front. Pharmacol..

[26] Kwiecień I., Smolin J., Beerhues L., Ekiert H. (2018). The impact of media composition on production of flavonoids in agitated shoot cultures of the three *Hypericum perforatum* L. cultivars ‘Elixir,’ ‘Helos,’ and ‘Topas’. Vitr. Cell. Dev. Biol. Plant.

[27] Radušienė J., Marksa M., Ivanauskas L., Jakštas V., Çalişkan Ö., Kurt D., Odabaş M.S., Çirak C. (2019). Effect of nitrogen on herb production, secondary metabolites and antioxidant activities of *Hypericum pruinatum* under nitrogen application. Ind. Crops Prod..

[28] Hernández-Saavedra D., Pérez-Ramírez I.F., Ramos-Gómez M., Mendoza-Díaz S., Loarca-Piña G., Reynoso-Camacho R. (2016). Phytochemical characterization and effect of Calendula officinalis, *Hypericum perforatum*, and *Salvia officinalis* infusions on obesity-associated cardiovascular risk. Med. Chem. Res..

[29] Oliveira A.I., Pinho C., Fonte P., Sarmento B., Dias A.C.P. (2018). Development, characterization, antioxidant and hepatoprotective properties of poly(ε-caprolactone) nanoparticles loaded with a neuroprotective fraction of *Hypericum perforatum*. Int. J. Biol. Macromol..

[30] Mohammed N.R., Dalar A., Ozdemir F.A., Turker M. (2019). In vitro propagation and secondary metabolites investigation in vitro propagation and secondary metabolites investigation of *Hypericum perforatum* L.. Fresenius Environ. Bull..

[31] Ersoy E., Eroglu Ozkan E., Boga M., Yilmaz M.A., Mat A. (2019). Anti-aging potential and anti-tyrosinase activity of three *Hypericum* species with focus on phytochemical composition by LC–MS/MS. Ind. Crops Prod..

[32] Zeljkovic S.C., Karalija E., Paric A., Muratovic E., Tarkowski P. (2017). Environmental factors do not affect the phenolic profile of *Hypericum perforatum* growing wild in Bosnia and Herzegovina. Nat. Prod. Commun..

[33] Margină D., Olaru O.T., Ilie M., Gradinaru D., GuTu C., Voicu S., Dinischiotu A., Spandidos D.A., Tsatsakis A.M. (2015). Assessment of the potential health benefits of certain total extracts from *Vitis vinifera*, *Aesculus hyppocastanum* and *Curcuma longa*. Exp. Ther. Med..

[34] Khemakhem I., Fuentes A., Lerma-García M.J., Ayadi M.A., Bouaziz M., Barat J.M. (2019). Olive leaf extracts for shelf life extension of salmon burgers. Food Sci. Technol. Int..

[35] Rizzo M., Ventrice D., Giannetto F., Cirinnà S., Santagati N.A., Procopio A., Mollace V., Muscoli C. (2017). Antioxidant activity of oleuropein and semisynthetic acetyl-derivatives determined by measuring malondialdehyde in rat brain. J. Pharm. Pharmacol..

[36] Micucci M., Malaguti M., Toschi T.G., Di Lecce G., Aldini R., Angeletti A., Chiarini A., Budriesi R., Hrelia S. (2015). Cardiac and vascular synergic protective effect of *Olea europea* L. leaves and *Hibiscus sabdariffa* L. flower extracts. Oxid. Med. Cell. Longev..

[37] Ammendola S., Cocchiola R., Lopreiato M., Politi L., Scandurra R., Giusti A.M., Scotto d’Abusco A. (2018). Olive fruit blends modulate lipid-sensing nuclear receptor PPARγ, cell survival, and oxidative stress response in human osteoblast cells. J. Am. Coll. Nutr..

[38] Kouka P., Priftis A., Stagos D., Angelis A., Stathopoulos P., Xinos N., Skaltsounis A.L., Mamoulakis C., Tsatsakis A.M., Spandidos D.A. (2017). Assessment of the antioxidant activity of an olive oil total polyphenolic fraction and hydroxytyrosol from a Greek *Olea europea* variety in endothelial cells and myoblasts. Int. J. Mol. Med..

[39] Leporini M., Loizzo M.R., Tenuta M.C., Falco T., Sicari V., Pellicanò T.M., Tundis R. (2018). Calabrian extra-virgin olive oil from *Frantoio cultivar*: Chemical composition and health properties. Emirates J. Food Agric..

[40] Kelebek H., Sevindik O., Selli S. (2019). LC-DAD-ESI-MS/MS-based phenolic profiling of St John’s Wort Teas and their antioxidant activity: Eliciting infusion induced changes. J. Liq. Chromatogr. Relat. Technol..

[41] Sevastre-Berghian A.C., Toma V.A., Sevastre B., Hanganu D., Vlase L., Benedec D., Oniga I., Baldea I., Olteanu D., Moldovan R. (2018). Characterization and biological effects of *Hypericum* extracts on experimentally-induced—Anxiety, oxidative stress and inflammation in rats. J. Physiol. Pharmacol..

[42] Napoli E., Siracusa L., Ruberto G., Carrubba A., Lazzara S., Speciale A., Cimino F., Saija A., Cristani M. (2018). Phytochemical profiles, phototoxic and antioxidant properties of eleven *Hypericum* species—A comparative study. Phytochemistry.

[43] Bonomo M.G., Cafaro C., Russo D., Calabrone L., Milella L., Saturnino C., Capasso A., Salzano G. (2018). Antimicrobial activity, antioxidant properties and phytochemical screening of *Aesculus hippocastanum* mother tincture against food-borne bacteria. Lett. Drug Des. Discov..

[44] Vašková J., Fejerčáková A., Mojžišová G., Vaško L., Patlevič P. (2015). Antioxidant potential of *Aesculus hippocastanum* extract and escin against reactive oxygen and nitrogen species. Eur. Rev. Med. Pharmacol. Sci..

[45] Amoo S.O., Aremu A.O., Moyo M., Van Staden J. (2012). Antioxidant and acetylcholinesterase-inhibitory properties of long-term stored medicinal plants. BMC Complement. Altern. Med..

[46] Altun M.L., Yilmaz B.S., Orhan I.E., Citoglu G.S. (2013). Assessment of cholinesterase and tyrosinase inhibitory and antioxidant effects of *Hypericum perforatum* L. (St. John’s wort). Ind. Crops Prod..

[47] Zengin G., Sarikurkcu C., Aktumsek A., Ceylan R., Ceylan O. (2014). A comprehensive study on phytochemical characterization of *Haplophyllum myrtifolium* Boiss. endemic to Turkey and its inhibitory potential against key enzymes involved in Alzheimer, Skin diseases and type II diabetes. Ind. Crops Prod..

